# Acute Effects of a High-Fat Meal Enriched with Pomegranate Seed Oil on Postprandial Lipemia and Endothelial Function in Postmenopausal Women: a Randomized Controlled Crossover Trial

**DOI:** 10.1016/j.tjnut.2026.101374

**Published:** 2026-01-22

**Authors:** Manal M Almoraie, Jeremy PE Spencer, Carol Wagstaff, Kim G Jackson

**Affiliations:** 1 Hugh Sinclair Unit of Human Nutrition, Department of Food and Nutritional Sciences, University of Reading, Whiteknights, Reading, United Kingdom; 2 Department of Food Science and Nutrition, Faculty of Human Sciences and Design, King Abdulaziz University, Jeddah, Saudi Arabia; 3 Institute for Cardiovascular and Metabolic Research and Institute of Food, Nutrition and Health, University of Reading, Reading, United Kingdom

**Keywords:** cell adhesion molecules, microvascular reactivity, postprandial lipemia, systolic blood pressure, triacylglycerol

## Abstract

**Background:**

Postprandial elevation of triacylglycerol (TAG) is associated with endothelial dysfunction and represents an important independent cardiovascular disease (CVD) risk factor in women. Although daily intakes of pomegranate seed oil (PSO, 80% conjugated α-linolenic acids) reduce fasting lipids, little is known about the acute effects on postprandial CVD risk markers.

**Objectives:**

This study compared the impact of a PSO-rich meal with those of a control meal on postprandial TAG (primary outcome measure), lipids, glucose, insulin, microvascular function, and cell adhesion molecule responses in postmenopausal women.

**Methods:**

In a single-blind, randomized controlled postprandial crossover study, 16 postmenopausal women aged ≤65 y were assigned to consume either a PSO-rich or a control meal on 2 separate occasions, 4 to 6 wk apart. A high-fat mixed meal (50 g fat) was provided at breakfast (0 min), and blood samples collected until 480 min postprandially to assess CVD risk markers. Specific time points were selected for blood pressure (BP) (0, 120, 240, 360 and 480 min) and microvascular reactivity (0, 180, 300, and 420 min). Postprandial data were analyzed using linear mixed models.

**Results:**

Compared with the control meal, the PSO-rich meal significantly reduced the postprandial TAG response but glucose, insulin, apolipoprotein B, and non-esterified fatty acid responses were similar. The AUC and incremental AUC (iAUC) for the postprandial acetylcholine (endothelium-dependent vasodilation) induced reactivity response were greater (*P* ≤ 0.04), and systolic BP lower after the PSO-rich meal than the control meal. Additionally, the iAUC for the pulse wave velocity and AUC/iAUC for the soluble intercellular adhesion molecule-1 responses were lower, whereas plasma nitrite concentrations were higher after the PSO-rich than control meal (*P* ≤ 0.037).

**Conclusions:**

A PSO-rich meal significantly reduced the postprandial TAG response and enhanced endothelial function compared with a control meal, suggesting a potential cardioprotective effect in postmenopausal women.

This study was registered at clinicaltrials.gov as NCT06042673 (https://clinicaltrials.gov/study/NCT06042673).

## Introduction

Cardiovascular diseases (CVD) are a significant concern in females transitioning to menopause [1]. Hormonal changes associated with this stage of life contribute to a higher CVD risk, driven by factors such as visceral obesity, atherogenic dyslipidemia, vascular dysfunction, and high blood pressure (BP). However, a direct association between menopause and increased risk of CVD events has only been confirmed in cases of early (premature) menopause [1,2]. Non-fasting triacylglycerol (TAG) concentrations are an independent, modifiable CVD risk factor [3,4], and considered more discriminatory of risk than fasting concentrations in women [[5], [6], [7]]. Findings from our studies, as well as those conducted by others, have shown postprandial TAG concentrations in response to high-fat meals to be greater in postmenopausal than premenopausal women [[8], [9], [10]], but findings are inconsistent [4]. In our previous study, discordance of the effects of age and menopausal status on the postprandial TAG response indicated that the decline in estrogen could potentially have unfavorable effects on pathways of TAG metabolism [8]. Exaggerated postprandial lipemia has often been linked with a temporary state of fat-induced oxidative stress, which reduces the bioavailability of the potent vasodilator nitric oxide (NO) [11], and transiently impairs vascular function. Since these short-term changes in vascular function are repeated on a daily basis and could have implications for long-term vascular health, the postprandial TAG response now represents an important therapeutic target. In particular, the amount, type, and dietary source of fat in a mixedmacronutrient meal have been shown to influence postprandial lipemia and vascular reactivity [12]. In general, meals containing higher doses of omega-3 (n–3) PUFA, particularly derived from fish oils, are associated with beneficial effects compared with those rich in SFA, MUFA, and n–6 PUFA [13], but findings are inconclusive due to the limited studies conducted [11]. Of those determining the effect of meals with varying fat composition on postprandial CVD risk markers, very few studies have been conducted in postmenopausal women, a population subgroup at higher CVD risk. In the study by Robertson et al. [14], a SFA-rich breakfast was associated with lower postprandial insulin sensitivity than meals rich in MUFA, n–6 PUFA, and a mixture of n–6 and n–3 PUFA, with little difference in the post-meal TAG responses between test oils. In agreement, Rathnayake et al. [15] found that sequential meals rich in SFA, MUFA, and n–6 PUFA had similar effects on postprandial TAG but opposing effects on BP and specific biomarkers of endothelial activation. Since substituting foods high in SFAs with unsaturated fatty acids is one of the most widely recognized dietary approaches for CVD prevention [[16], [17], [18]], further studies are needed to evaluate the impact of meal fat composition on postprandial CVD markers to address health inequalities in postmenopausal women [19].

Pomegranate extracts and products are widely consumed for their reported cardioprotective effects [[20], [21], [22]], with increasing reports of health benefits during menopause [23,24]. Long-term intakes of pomegranate products (juice, extract, and seed oil) can improve the fasting lipid profile and BP. However, the efficacy of pomegranate seed oil (PSO) on CVD and its risk factors remains unclear, partly because PSO doses and extraction methods have varied across studies [[25], [26], [27], [28]]. PSO is particularly rich in the PUFA punicic acid (*cis*-9, *trans*-11, and *cis*-13 18:3), a conjugated α-linolenic acid (CLnA) [29], which is thought to have more potent hypolipidemic effects than ordinary CLnAs [30]. Although individuals spend a large proportion of the day in the fed state, no studies to date have determined the effects of this seed oil on CVD risk markers during the postprandial phase [21]. Therefore, this study aimed to investigate the effects of a PSO-rich meal on the postprandial serum TAG response (primary outcome measure) and lipemia-induced endothelial dysfunction in postmenopausal women. We hypothesized that consumption of a high-fat PSO-rich meal would attenuate postprandial increases in serum TAG and reduce lipemia-induced endothelial dysfunction compared with a control meal.

## Methods

### Participants

This single-center study was conducted at the Hugh Sinclair Unit of Human Nutrition, University of Reading (United Kingdom), between June 2023 and February 2024. Sixteen postmenopausal women (aged ≤65 y; BMI: 18–35 kg/m^2^) were recruited from the University of Reading and the surrounding areas using inclusion/exclusion criteria that aligned with our previous study conducted in this subject group [15]. Interested individuals were provided with a participant information sheet and instructed to complete a medical and lifestyle questionnaire to determine their eligibility. Those who met the preliminary criteria were invited to a brief screening visit, during which written and verbal informed consent was obtained. The eligibility criteria included being female, postmenopausal (self-reported, with no menstruation for ≥1 y), aged ≤65 y, a non-smoker, and consuming no more than the recommended alcohol intake (<14 units/wk, self-reported). Additional requirements included a BMI between 18 and 35 kg/m^2^, BP ≤140/90 mm Hg, no diagnosis of diabetes (fasting glucose levels <7.0 mmol/L), total cholesterol (TC) concentrations <7.5 mmol/L, TAG concentrations <2.3 mmol/L, normal liver and kidney biochemistry, and no anemia (hemoglobin ≥115 g/L). Exclusion criteria included a self-reported history of myocardial infarction or stroke in the past 12 mo, diagnosis of cardiovascular, respiratory, renal, gastrointestinal, cancer, or hepatic diseases; or current use of medications for hyperlipidemia, hypertension, inflammation, or hypercoagulation. Additional exclusions included hormone replacement therapy, a vegan diet, participation in weight-loss programs, use of nutritional supplements, and engaging in vigorous-intensity aerobic exercise more than 3 times/wk for ≥30 min/session.

### Study design

This was an acute, randomized, single-blind, placebo-controlled, crossover study in which postmenopausal women were randomly assigned to a sequence to receive the high-fat breakfast containing either a PSO-rich or a control oil on separate occasions. Participants were randomly allocated without stratification using Research Randomizer (http://www.randomizer.org/form.htm) by a single researcher (MMA). Only participants were blinded to the type of oil consumed at each study visit because this was incorporated into a warm chocolate drink so the test breakfast meals appeared similar. Each postprandial visit lasted ∼600 min (10 h) and was conducted on separate occasions, 4 to 6 wk apart. This wash-out period was in accordance with our previous postprandial studies in which test fats rich in SFA and PUFA (n–3 and n–6) were incorporated [[31], [32], [33]]. The study design and protocol have been previously used by the researchers in postmenopausal women [14,15], and found to be well tolerated by the participant group. The primary outcome was the postprandial TAG response, assessed by the time-course profile, AUC, and incremental AUC (iAUC). Secondary outcomes included BP, peripheral microvascular function [assessed using laser Doppler imaging with iontophoresis (LDI)], arterial stiffness [measured using pulse wave velocity (PWV)], insulin, glucose, apolipoprotein (apo)B, non-esterified fatty acids (NEFA), and markers of endothelial activation. A favorable ethical opinion for conduct was given by the University of Reading Research Ethics Committee (UREC 22/11), and the study was registered on clinicaltrials.gov (NCT06042673).

### Postprandial test meal composition

The breakfast test meal included a toasted sandwich made with white bread (100 g, equivalent to 2 thick slices, Hovis Ltd.), and strawberry seedless jam (30 g, Hartley’s, Histon Sweet Spreads Limited), and a warm chocolate drink [skimmed milk (150 g, Arla Foods United Kingdom Ltd.), Nesquik chocolate-flavored powder (15 g, Nestlé United Kingdom Ltd.), Marvel skimmed milk powder (15 g, Premier Foods Group Ltd.)] containing 50 g of the specific test fats. For the control oil, a mixture of palm oil (Sime Darby Oils), rapeseed oil (Sime Darby Oils), and safflower oil (Spectrum Culinary) in a 4:0.5:0.5 ratio was used to mimic the fat composition of a typical United Kingdom diet. The PSO-rich meal contained 40 g of the same oil mixture and 10 g of PSO (Pödör USA Inc.). The nutrient composition of the breakfast meals containing the different test fats is presented in Table 1, whereas the fatty acid composition of the PSO and control oils is presented in Supplemental Table 1.

**TABLE 1:** Energy and macronutrient composition of the pomegranate seed oil-rich and control test meals^1^

	Pomegranate seed oil-rich meal	Control meal
Energy (MJ)	4.07	4.07
Fat (g)	52.3	52.3
SFAs	18.2	22.0
MUFAs	15.7	18.8
PUFAs	16.3	9.3
n–6 PUFA	7.5	8.7
n–3 PUFA	0.48	0.60
Punicic acid	8.3	—
Carbohydrate (g)	90.4	90.4
Sugars (g)	43.9	43.9
Protein (g)	21.3	21.3

1 Macronutrient composition from the manufacturers’ data.

### Study visits

During the screening visit, various measurements were performed to assess eligibility. Height was recorded to the nearest 0.5 cm using a wall-mounted stadiometer. Weight was measured using the Tanita BC-418 scale (Tanita Europe) under the standard body typesetting, and BMI was calculated. BP was measured 3 times using an OMRON M6 automatic digital BP monitor (OMRON). Furthermore, a 12-h fasted blood sample (9 mL) was collected to evaluate fasting TC, TAG, glucose, and markers of kidney and liver function using the Randox Daytona+ clinical chemistry analyzer (Randox Laboratories Ltd.). Participants were assessed for anemia by measuring hemoglobin using a DxH 520 hematology analyzer (Beckman Coulter). Participants completed a 3-d unweighed food diary (1 weekend day and 2 week days) to evaluate their habitual dietary intake during the 1-wk period before each study visit. The data were analyzed using Nutritics nutrient analysis software (Nutritics, 2023).

Due to the intra-individual variability in the postprandial TAG response, participants were required to maintain their usual diet and physical activity levels between study visits. For 24 h before each study visit, instructions were given to refrain from consuming alcohol and engaging in aerobic exercise, and a pre-prepared low-fat evening meal (containing <10 g of total fat) was provided to standardize short-term fat intake. After a 12-h fast, participants arrived at the clinical unit of the Hugh Sinclair Unit of Human Nutrition, having consumed only low-nitrate water (Buxton Mineral Water) overnight and on the morning of the visit. Postprandial study visits (lasting 600 min) were conducted in a temperature-controlled clinical room maintained at 22 ± 1°C. After assessing body composition, a cannula was inserted into the antecubital vein of the forearm to facilitate frequent blood sampling. After the baseline fasting blood draw (–30 min) and a 30-min rest period in the supine position, endothelial function was assessed using LDI. A second baseline blood sample was collected (time 0) before the participants were served a standardized breakfast test meal, which was consumed within 20 min. Subsequent blood samples were collected at regular intervals: every 30 min until 120 min and then every 60 min until 480 min. LDI assessments were performed at baseline, 180, 300, and 420 min. BP and pulse wave analysis (PWA) were performed at baseline and 120, 240, 360, and 480 min post-meal. Participants remained in the clinical unit for the entire duration of their visit and no other food or drink except ad-libitum water was allowed throughout the study day. To mitigate any effects of the amount and timing of water consumption on the postprandial lipemic response, ad-libitum water intake was recorded on the first study visit and matched during the subsequent visit.

### Sample analyses

Blood samples were collected into either lithium heparin-coated tubes for plasma or serum separator tubes (VACUETTE; Greiner Bio-One). Plasma samples were briefly stored on ice, whereas serum samples were left to clot for up to 30 min at room temperature. Both blood tubes were then centrifuged at 1700 × *g* for 15 min at 4°C. Plasma samples were stored at –80°C, whereas serum samples were kept at –20°C until analysis.

The outcomes measured in blood samples collected at all time points (–30, 0, 30, 60, 90, 120, 180, 240, 300, 360, 420, and 480 min) included serum TAG, NEFA, insulin, and glucose. Serum apoB was analyzed in samples collected at 0, 60, 120, 180, 240, 300, 360, 420, and 480 min, whereas plasma nitrite, nitrate, and markers of endothelial activation were measured at 0, 180, 300, and 420 min (time points which aligned with the LDI measurements). TC, HDL-cholesterol and C-reactive protein were measured in fasting samples only (–30 and 0 min) on each study visit.

Serum concentrations of TAG, apoB, NEFA, glucose, HDL-cholesterol, TC, and C-reactive protein were measured using a Randox Daytona+ analyzer. Plasma nitrite and nitrate concentrations were analyzed using HPLC, as previously described [34]. Serum insulin and plasma soluble vascular cell adhesion molecule (sVCAM)-1, soluble intercellular adhesion molecule (sICAM)-1, E-selectin, and P-selectin were quantified using Simple Plex cartridges on the Ella automated ELISA platform (Bio-Techne). Analyses were performed after all study visits were completed, and all samples for a given participant were processed in a single batch.

Data collected during the screening visit were used to estimate 10-y CVD risk using the QRISK 3-2018 online tool (http://www.qrisk.org/index.php). Estimates of insulin resistance and sensitivity were calculated using the homeostatic model assessment for insulin resistance and the revised quantitative insulin sensitivity check index, respectively. The TC:HDL-cholesterol ratio was calculated and the LDL-cholesterol concentration in the 0 min (baseline) sample was estimated using the Friedewald formula.

### Assessment of vascular function and BP

Participants rested in a supine position for 30 min in a quiet, temperature-controlled environment (22 ± 1°C) before vascular function assessments. LDI was conducted using an LDI2-IR laser Doppler imager (Moor Instruments Ltd.), as previously described [35]. Microvascular responses to 1% acetylcholine (ACh; endothelium-dependent vasodilation) and 1% sodium nitroprusside (SNP; endothelium-independent vasodilation) were assessed on the volar surface of the left forearm using transdermal iontophoresis (MIC2, Moor Instruments Ltd.). These responses were quantified as the AUC of flux, measured in arbitrary perfusion units over time, using a 21-scan protocol. All measurements were performed by 1 researcher (MMA) to minimize interoperator variability, and the within-day and between-day coefficients of variation for this method were both <10% [36].

PWV, systolic BP (SBP), and diastolic BP (DBP) were measured in duplicate from the left arm using an oscillometric cuff connected to a PWA device (Mobil-O-Graph).

### Statistical analyses

As no previous studies had determined the effects of PSO on postprandial lipemia, the sample size calculation was based on expected differences in incremental postprandial TAG responses between SFAs and unsaturated fatty acids, using data from the systematic review and meta-analysis of Monfort-Pires et al. [37]. The analysis predicted a mean TAG difference of 67.7 mmol/L × min, with a SD of 47.0. To achieve 90% power at a 5% significance level, 13 participants were required. The total sample size was increased to 16 to allow for a 20% dropout rate.

All statistical analyses were conducted using IBM SPSS Statistics version 27. Data were assessed for normality and log-transformed where necessary. The AUC and iAUC were calculated over the 0 to 480 min period for serum TAG, apoB, glucose, and insulin responses (variables with ≥11 time points). The AUC and iAUC calculations for NEFA were limited to the 180 to 480 min post-breakfast period to account for the drop in NEFA concentrations that occurs during the initial 90 to 180 min after meal ingestion. These values were calculated from the mean minimum NEFA concentration.

Baseline characteristics before the PSO and control study visits were analyzed using paired *t*-tests for continuous variables. Postprandial time-course responses to the test meals were evaluated for all primary and secondary outcomes using a linear mixed-model analysis (PROC MIXED). The initial models included test oil × time interaction effects, which were retained if significant. Since age and BMI have both been shown to have independent effects on vascular function and postprandial lipemia, fixed effects (period, time, treatment, age, and BMI) and random effects (participant) were included in all linear mixed models, regardless of their statistical significance levels. Results were deemed significant at *P* ≤ 0.05 for both the primary (postprandial TAG response) and secondary outcome measures.

## Results

### Study participation

**FIGURE 1:**
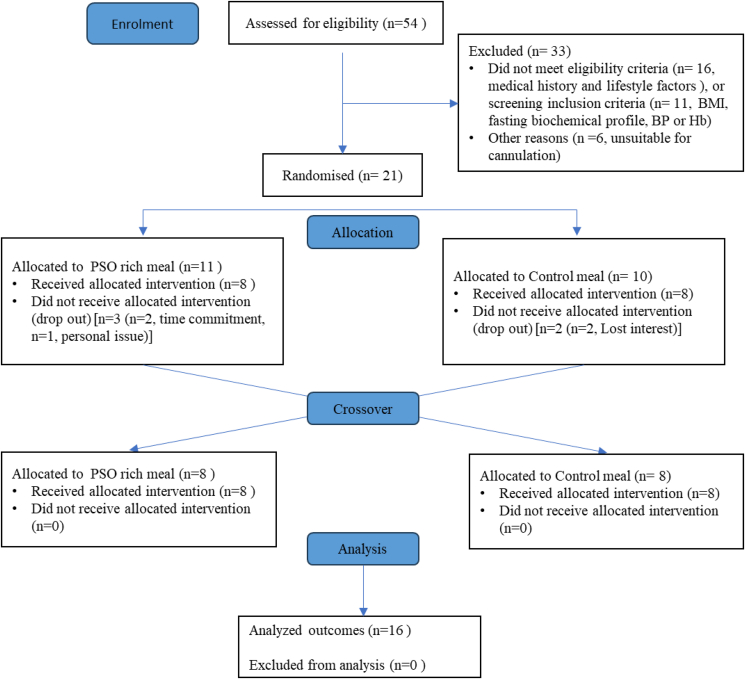
Participant inclusion flowchart for the acute PSO study. BP, blood pressure; Hb, hemoglobin; PSO, pomegranate seed oil.

Of the 54 volunteers assessed for eligibility to participate in the study using the initial questionnaire, 21 were randomly assigned to a test oil sequence, and 5 withdrew due to lack of time commitment (*n* = 2), loss of interest (*n* = 2), and personal reasons (*n* = 1). A total of 16 postmenopausal females completed both study visits, and data were available for all participants for each of the outcomes (Figure 1). These participants had a mean ± SEM age of 59 ± 1 y (range 49–64 y) and a BMI of 24.8 ± 0.9 kg/m^2^ (*n* = 9 had a BMI in the normal weight range and *n* = 7 in the overweight BMI category). At screening, the average CVD risk score of the participants, calculated using the QRISK3 tool, was 4.9% ± 0.5% indicative of a low CVD risk. The estimate of insulin sensitivity, assessed by the revised quantitative insulin sensitivity check index was 0.45 ± 0.02, and the HOMA-IR was 1.3 ± 0.3, both considered in the normal range for healthy individuals. Participant characteristics (anthropometric measures, biochemical profile and habitual diet) and outcomes measured in the fasting (baseline) samples were not different between study visits and the mean values for the 2 study visits are shown in Table 2. (Supplemental Table 2 presents the participant characteristics and selected baseline outcomes at the beginning of the control and PSO-rich meal acute study sessions**,** with fasting data for the serum lipid, glucose, and insulin responses presented in Table 3 and BP and PWV in Table 4.) All participants consumed the test meals in full without any adverse events.

**TABLE 2:** Mean baseline characteristics of the participants^1^

Characteristics	Mean values	Range
Body weight (kg)	67.6 ± 3.3	50–94
BMI (kg/m^2^)	24.7 ± 0.9	20.7–29.7
Waist circumference (cm)	88.8 ± 2.3	74–103
Hip circumference (cm)	103 ± 2	92–121
Body fat (%)	35.2 ± 1.7	22.5–45.0
Blood pressure (mmHg)		
Systolic	121 ± 3	101–143
Diastolic	80 ± 2	64–94
PWV (m/s)	8.1 ± 0.2	6.6–9.4
Fasting serum biochemical profile		
TC (mmol/L)	5.40 ± 0.18	4.60–6.65
LDL-C (mmol/L)	3.33 ± 0.16	2.76–4.60
HDL-C (mmol/L)	1.51 ± 0.07	1.04–2.12
TC:HDL ratio	3.69 ± 0.22	2.90–6.30
TAG (mmol/L)	1.17 ± 0.10	0.54–2.24
ApoB (g/L)	0.98 ± 0.05	0.64–1.47
C-reactive protein (mg/L)	1.54 ± 0.36	0.10–6.30
Glucose (mmol/L)	4.69 ± 0.13	4.08–5.82
Insulin (pmol/L)	37.7 ± 3.5	17.8–64.0
NEFA (mmol/L)	0.51 ± 0.04	0.29–0.98
Habitual macronutrient intake		
Energy (MJ/d)	7.2 ± 0.4	4.8–11.0
Total fat (%TE)	36.1 ± 1.4	27.5–46.9
SFAs (%TE)	14.1 ± 0.7	8.7–17.4
MUFAs (%TE)	12.8 ± 0.9	0.9–14.4
n–6 PUFAs (%TE)	5.5± 0.4	0.2–7.2
n–3 PUFAs (%TE)	0.7 ± 0.1	0.1–1.7
TFAs (%TE)	0.5 ± 0.0	0.4–0.6
Dietary cholesterol (mg/d)	154 ± 15	148–206
Protein (%TE)	13.6 ± 0.7	8.3–19.8
Carbohydrates (%TE)	45.6 ± 1.3	37.1–54.9
Dietary fiber (AOAC) (g/d)	25.5 ± 2.7	10.3–49.0
Alcohol (%TE)	2.1 ± 0.7	0.0–7.7

Abbreviations: AOAC, Association of Analytical Chemists; Apo, apolipoprotein; NEFA, non-esterified fatty acids; PSO, pomegranate seed oil; PWV, pulse wave velocity; TAG, triacylglycerol; TC, total cholesterol; TE, total energy; TFA, *trans* fatty acid.

1 Values are expressed as means ± SEMs and ranges; *n* = 16 for all outcomes. The dietary data were collected using a 3-d unweighed food diary and mean nutrient intakes determined using Nutritics.

**TABLE 3:** Fasting concentrations and postprandial summary measures for the serum lipid, glucose, and insulin responses in postmenopausal females after acute consumption of the pomegranate seed oil-rich and control meals^1^

	PSO-rich meal	Control meal	*P*^2^
TAG			
Fasting (mmol/L)	1.15 ± 0.09	1.18 ± 0.11	0.640
MaxC (mmol/L)	1.91 ± 0.13	2.24 ± 0.14	0.019
TMax^3^ (min)	248 ± 29	338 ± 19	0.021
AUC (mmol/L × min)	727 ± 49	825 ± 59	0.030
iAUC (mmol/L × min)	176 ± 20	257 ± 27	0.005
ApoB			
Fasting^3^ (g/L)	0.98 ± 0.05	0.97 ± 0.05	0.540
MaxC^3^ (g/L)	1.05 ± 0.05	1.06 ± 0.05	0.836
TMax^2^ (min)	214 ± 42	296 ± 40	0.057
AUC^3^ (g/L × min)	470 ± 25	477 ± 21	0.717
iAUC (g/L × min)	–0.78 ± 3.41	9.33 ± 5.92	0.120
NEFA			
Fasting (mmol/L)	0.50 ± 0.04	0.51 ± 0.04	0.770
MinC^3^ (mmol/L)	0.11 ± 0.01	0.12 ± 0.01	0.080
TMin^3^ (min)	187 ± 14	169 ± 16	0.290
Suppression (%)	–77 ± 2	–74 ± 3	0.160
MaxC (mmol/L)	0.83 ± 0.06	0.86 ± 0.05	0.810
TMax (min)	345 ± 44	364 ± 37	0.830
AUC_180–480_ (mmol/L × min)	142 ± 13	153 ± 12	0.410
iAUC^3^_180–480_ (mmol/L × min)	–7.4 ± 14.6	–0.4 ± 15.7	0.630
Glucose			
Fasting (mmol/L)	4.64 ± 0.12	4.74 ± 0.13	0.080
MaxC (mmol/L)	6.07 ± 0.23	6.53 ± 0.28	0.075
TMax (min)	124 ± 15	143 ± 24	0.749
AUC (mmol/L × min)	2333 ± 46	2363 ± 67	0.603
iAUC (mmol/L × min)	104 ± 52	89 ± 49	0.679
Insulin			
Fasting (pmol/L)	37.3 ± 3.3	38.1 ± 3.7	0.690
MaxC (pmol/L)	427 ± 48	432 ± 60	0.873
TMax (min)	113 ± 12	105 ± 7	0.521
AUC (nmol/L × min)	66.0 ± 6.3	65.0 ± 6.7	0.805
iAUC (nmol/L × min)	48.2 ± 5.3	46.7 ± 5.2	0.700

Abbreviations: Apo, apolipoprotein; iAUC, incremental AUC; MaxC, maximum concentration; MinC, minimum concentration; NEFA, non-esterified fatty acids; PSO, pomegranate seed oil; TAG, triacylglycerol; TMax, time to reach maximum concentration; TMin, time to reach minimum concentration.

1 Values are expressed as means ± SEMs for the fasting concentrations and unadjusted means ± SEMs for the postprandial summary measures, *n* = 16 for all outcomes. Unless otherwise specified, the time interval between the AUC and iAUC responses was 480 min.

2 Fasting concentrations were analyzed using an unpaired *t*-test. Linear mixed-model analyses were used to determine the overall treatment effect on postprandial summary measures, with adjustments made for the fixed effects of period, treatment, age, and BMI. Participant was included as a random effect. *P* ≤ 0.05 was considered statistically significant.

3 Indicates data were log-transformed before analysis.

**TABLE 4:** Fasting concentrations and postprandial summary measures for the vascular outcomes, blood pressure, and endothelial activation marker responses in postmenopausal females after acute consumption of the pomegranate seed oil-rich and control meals^1^

	PSO-rich meal	Control meal	*P* value^2^
Vascular function			
LDI			
LDI-Ach			
Fasting (PU)	979 ± 58	967 ± 44	0.810
AUC (PU × min)	500,883 ± 29,086	415,464 ± 21,806	0.040
iAUC^3^ (PU × min)	89,835 ± 20,435	9324 ± 19,957	0.009
LDI-SNP			
Fasting^3^ (PU)	1143 ± 124	1166 ± 153	0.470
AUC^3^ (PU × min)	1451 ± 130	1467 ± 138	0.880
iAUC (PU × min)	483 ± 112	547 ± 131	0.801
PWV			
Fasting (m/s)	8.26 ± 0.19	7.99 ± 0.19	0.080
AUC (m/s × min × 10^3^)	3.9 ± 0.08	3.9 ± 0.08	0.960
iAUC (m/s × min × 10^3^)	–0.05 ± 0.03	0.75 ± 0.03	<0.001
Blood pressure			
SBP			
Fasting (mmHg)	122 ± 3	119 ± 3	0.340
AUC (mmHg × min × 10^3^)	56.9 ± 1.4	59 ± 1.5	0.050
iAUC (mmHg × min × 10^3^)	–1.6 ± 0.7	1.7 ± 0.8	0.002
DBP			
Fasting (mmHg)	79 ± 2	80 ± 2	0.620
AUC (mmHg × min × 10^3^)	36.9 ± 1.1	38.4 ± 1.1	0.004
iAUC (mmHg × min × 10^3^)	–1.0 ± 0.7	0.2 ± 0.5	0.078
Circulating plasma markers of endothelial activation			
Nitrite^3^			
Fasting (μmol/L)	0.26 ± 0.06	0.24 ± 0.03	0.897
AUC (μmol/L × min)	122.5 ± 29.9	94.8 ± 10.4	0.642
iAUC (μmol/L × min)	15.8 ± 8.5	–5.2 ± 6.4	0.379
Nitrate			
Fasting (μmol/L)	31.5 ± 2.5	33.9 ± 3.1	0.390
AUC (μmol/L × min)	11,248 ± 940	11,260 ± 1176	0.988
iAUC (μmol/L × min)	–1967 ± 490	–3001 ± 416	0.101
sVCAM-1			
Fasting (μg/L)	450 ± 22	453 ± 26	0.866
AUC (g/L × min)	187 ± 8.9	192 ± 9.8	0.552
iAUC (g/L × min)	–1.39 ± 2.91	1.70 ± 3.70	0.535
sICAM-1			
Fasting (μg/L)	253 ± 10	253 ± 9	0.920
AUC (g/L × min)	97.7 ± 3.8	104.5 ± 3.4	0.037
iAUC (g/L × min)	–8.54 ± 1.38	–2.05 ± 1.75	0.004
E-selectin			
Fasting (μg/L)	29.2 ± 2.4	28.3 ± 2.1	0.150
AUC (g/L × min)	11.9 ± 0.9	12.1 ± 0.9	0.494
iAUC (g/L × min)	0.24 ± 0.16	0.21 ± 0.11	0.583
P-selectin			
Fasting (μg/L)	63.1 ± 4.5	64.3 ± 4.5	0.457
AUC (g/L × min)	26.4 ± 1.7	26.4 ± 1.9	0.985
iAUC^3^ (g/L × min)	–0.28 ± 0.78	–0.63 ± 0.32	0.796

Abbreviations: ACh, acetylcholine; DBP, diastolic blood pressure; iAUC, incremental AUC; LDI, laser doppler imaging; PSO, pomegranate seed oil; PU, perfusion unit; PWV, pulse wave velocity; SBP, systolic blood pressure; sICAM, soluble intercellular adhesion molecule; SNP, sodium nitroprusside; sVCAM, soluble vascular cell adhesion molecule.

1 Values are expressed as means ± SEMs for the fasting data and unadjusted means ± SEMs for the postprandial summary measures, *n* = 16 for all outcomes. The time interval for the AUC and iAUC: 420 min for LDI and circulating markers of endothelial activation; 480 min for blood pressure and PWV.

2 Fasting data were analyzed using unpaired *t*-tests. Linear mixed-model analyses were performed to determine the overall treatment effect in postprandial summary measures, with adjustments made for fixed effects of period, treatment, age, and BMI. Participant was included as a random effect. *P* ≤ 0.05 was considered statistically significant.

3 Indicates data were log-transformed before analysis.

### Postprandial lipid, glucose, and insulin response

**FIGURE 2:**
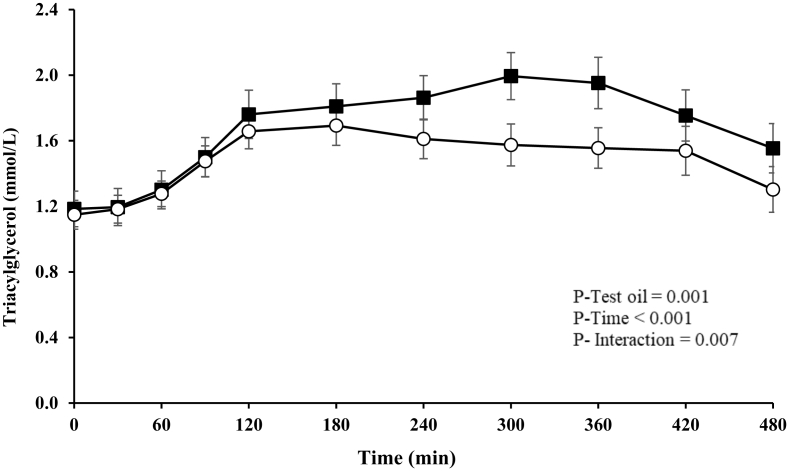
Postprandial serum triacylglycerol responses in postmenopausal females after consumption of the pomegranate seed oil-rich (open circles) and control (closed squares) meals. Values are expressed as unadjusted means ± SEMs, *n* = 16. Linear mixed-model analysis was used to explore the effects of treatment and time, with an adjustment made in all cases for fixed effects of period, time, treatment, age, and BMI. The participant was included as a random effect. *P* ≤ 0.05 was considered statistically significant.

A significant effect of test oil (*P* = 0.001) and a test oil × time interaction (*P* = 0.007) were observed for the postprandial TAG response (Figure 2), with a lower AUC (*P* = 0.030), iAUC (*P* = 0.005), and maximum TAG concentration (*P* = 0.019) reached after the PSO-rich meal compared with the control meal. Additionally, the time to reach the maximum concentration was also found to be, on average, 90 min later after the control meal than after the PSO-rich meal (*P* = 0.021) (Table 3). Postprandial NEFA, apoB, glucose, and insulin responses or postprandial summary measures did not differ between the PSO-rich and control meals, as presented in Table 3.

### Postprandial vascular function response

Fasting and postprandial summary measures for the skin perfusion responses to iontophoresis of ACh and SNP are presented in Table 4. A significant test oil effect (*P* < 0.001) only was observed for the ACh vasodilatory response (i.e., the area under the flux compared with time curve) with a trend for an increase after the PSO-rich meal associated with a greater AUC (*P* = 0.040) and iAUC (*P* = 0.009) compared with that in the control meal (Supplemental Figure 1A and Table 4). The SNP vasodilatory responses were not found to be different between the test oils (test oil effect *P* = 0.740; AUC, *P* = 0.880, and iAUC, *P* = 0.801) (Supplemental Figure 1B and Table 4).

### Postprandial BP and arterial stiffness response

**FIGURE 3:**
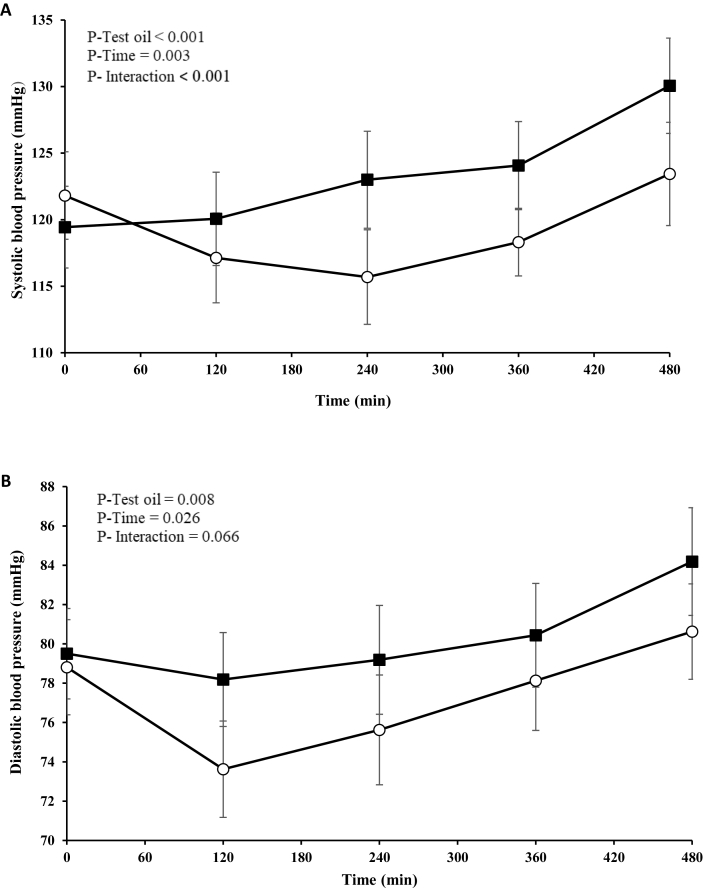
Postprandial (A) systolic blood pressure and (B) diastolic blood pressure responses in postmenopausal females after consumption of the pomegranate seed oil-rich (open circles) and control (closed squares) meals. Values are expressed as unadjusted means ± SEMs, *n* = 16. Linear mixed-model analysis was used to explore the effects of treatment and time, with an adjustment made in all cases for fixed effects of period, time, treatment, age, and BMI. Participant was included as a random effect. *P* ≤ 0.05 was considered statistically significant.

A significant test oil effect (*P* < 0.001) and test oil × time interaction (*P* < 0.001) were observed for the postprandial SBP response (Figure 3A), with a significantly lower AUC (*P* = 0.050) and iAUC (*P* = 0.002) found after consumption of the PSO-rich meal compared with the control meal (Table 4). For the postprandial DBP response, there was only a significant effect of the test oil (*P* = 0.008) (Figure 3B**)**, with a lower AUC (*P* = 0.004) observed after the PSO-rich meal compared with the control meal (Table 4). In terms of the postprandial PWV response, only the iAUC differed significantly between the test oils, which was lower after the PSO-rich meal than the control meal (*P* < 0.001) (Table 4).

### Postprandial nitrite and nitrate responses

**FIGURE 4:**
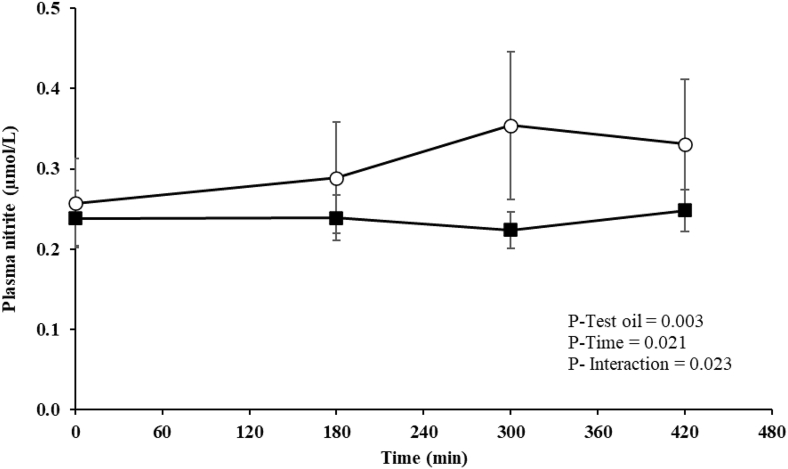
Postprandial plasma nitrite responses in postmenopausal females after consumption of the pomegranate seed oil-rich (open circles) and control (closed squares) meals. Values are expressed as unadjusted means ± SEMs, *n* = 16. Linear mixed-model analysis was used to explore the effects of treatment and time, with an adjustment made in all cases for fixed effects of period, time, treatment, age, and BMI. The participant was included as a random effect. *P* ≤ 0.05 was considered statistically significant.

Fasting nitrate and nitrite concentrations were not different before the consumption of the control and PSO-rich test meals (Table 4). A significant test oil effect (*P* = 0.003) and test oil × time interaction (*P* = 0.023) were observed for the postprandial plasma nitrite, but not for the nitrate response, with higher nitrite concentrations evident after the PSO-rich compared with that in the control meal (Figure 4). The postprandial summary measures (AUC and iAUC) for nitrite and nitrate concentrations did not differ between the test oils (Table 4).

### Postprandial response for markers of endothelial activation

**FIGURE 5:**
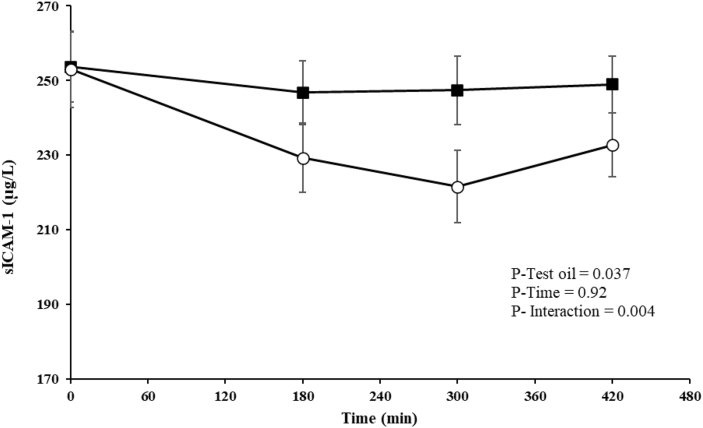
Postprandial plasma sICAM-1 response in postmenopausal females after consumption of the pomegranate seed oil-rich (open circles) and control (closed squares) meals. Values are expressed as unadjusted means ± SEMs, *n* = 16. Linear mixed-model analysis was used to evaluate the effects of treatment and time, with adjustments made for fixed factors, including period, time, treatment, age, and BMI. Participant was included as a random effect. *P ≤* 0.05 was considered statistically significant. sICAM, soluble intercellular adhesion molecule.

Concentrations of the markers of endothelial activation were similar in the baseline (fasting) sample (Table 4). A significant test oil effect (*P* = 0.037) and test oil × time interaction (*P*=0.004) were found for the postprandial sICAM-1 responses (Figure 5), with a lower AUC (*P* = 0.037) and iAUC (*P* = 0.004) following the PSO-rich meal compared with the control meal (Table 4). Postprandial plasma sVCAM-1, P-selectin, and E-selectin responses and summary measures were similar following the test oils (Table 4).

## Discussion

To the best of our knowledge, this is the first randomized controlled trial conducted to investigate the acute effects of a PSO-rich meal on postprandial lipemia and vascular function in postmenopausal females. Our study showed favorable effects of the PSO-rich meal on the postprandial TAG response, endothelium-dependent microvascular reactivity, BP, plasma nitrite, and sICAM-1 (a marker of endothelial activation), with minimal impact on the other outcomes measured.

Since individuals spend the majority of the day in the postprandial state, non-fasting TAG concentrations are now recognized as an important therapeutic target for CVD risk reduction. To date, most studies examining the effects of PSO on lipid metabolism have been chronic in design [21], with a daily intake of 800 mg reported to reduce fasting TAG [26,27]. Consistent with these findings, we observed that the PSO-rich meal lowered the postprandial TAG response (which supports our research hypothesis) and reduced the timing of the peak concentration compared with the control meal. Although acute studies specifically using PSO-rich meals are not available for comparison, our findings align with those of a systematic review and meta-analysis that reported lower TAG IAUC in studies over 8 h following PUFA compared with SFA-rich meals [37]. The amount, type, and dietary source of meal fatty acids are important factors in relation to the magnitude and duration of the lipemic response. This may explain the discrepancies with findings from 2 previous acute studies conducted in postmenopausal females which observed similar postprandial TAG responses to meals with varying fat compositions. In the study of Robertson et al. [14], the SFA-rich meal contained a mixture of palm oil and cocoa butter, whereas the PUFA-rich meals contained either safflower oil or a 50:50 mixture of safflower oil and long-chain n–3 PUFA. Rathnayake et al. [15] compared butter as a fat source in the SFA-rich oil with safflower oil in the n–6 PUFA-rich meal. In the current study, the PSO used was rich in CLnA but studies determining the postprandial responses to this class of fatty acids or punicic acid are limited [38]. Compared with SFA-rich meals, those high in n–3 PUFA, particularly fish oils, are generally associated with a more rapid rate of gastric emptying [14], and enhanced TAG-rich lipoprotein clearance, but the effects of plant-derived n–3 PUFA are considered less potent [39]. Furthermore, studies that focus on possible determinants of TAG-rich lipoprotein metabolism, and which include the measurement of postprandial plasma fatty acid compositions are now needed to gain insights into the mechanisms underlying the lower TAG response after the PSO-rich oil meal.

In the current study, the favorable effects of the PSO-rich meal on endothelium-dependent microvascular reactivity (an indicator of overall endothelial function) were consistent with a higher plasma nitrite response, a surrogate marker of bioavailable NO. Pomegranate products (including PSO) have been shown to enhance NO bioavailability and improve arterial function in obese Zucker rats, an animal model of the metabolic syndrome [40]. Ex vivo studies using rat aortic rings further showed that incubation with punicic acid promoted endothelium-dependent vasorelaxation, primarily by influencing the NO–guanylyl cyclase pathway [41], but that this effect was lost following the removal of the endothelial layer. These findings align with our observation that the PSO-rich meal promoted beneficial effects on ACh- but not SNP-induced reactivity, an endothelial-independent vasodilator. However, we do need to be cautious with this interpretation since the findings relate to an animal study incorporating only a single fatty acid and not a mixture as given in our breakfast meal. Improvements in endothelial function are likely mechanisms contributing to BP regulation and maintenance of vascular homeostasis. In agreement with a systematic review and meta-analysis of randomized controlled trials [22], small but significant reductions in postprandial SBP and to a lesser DBP were observed after the PSO-rich meal and found to be consistent with a lower PWV, a proximal measure of arterial stiffness. To date, only 1 short-term chronic study has examined the effects of pomegranate juice on PWV and found daily consumption to have modest beneficial effects on BP and mean arterial pressure but not on PWV [42]. Although very limited data are available, the type of pomegranate products consumed may be important in relation to the vascular benefits observed.

Endothelial dysfunction, a specific activated state of endothelial cells, is marked by increased expression and release of inflammatory cytokines including adhesion molecules (e.g., sICAM-1 and sVCAM-1), and cell surface adhesion molecules (e.g., P-selectin and E-selectin) into the bloodstream. Compared with the control meal, a lower postprandial AUC and iAUC for the sICAM-1 response was evident after the PSO-rich meal, while having minimal impact on other adhesion molecules. These findings are consistent with those reported by Rathnayake et al. [15] and Masson and Mensink [43], where postprandial ICAM-1 was lower after n–6 PUFA compared with SFA-rich meals. However, they contrast with the results of Stonehouse et al. [44], who observed no changes in inflammatory markers after comparing a high protein, fat-rich meal containing palmolein (a similar fat composition to our control meal) with MUFA-rich olive oil. Findings from in vitro studies have shown that the type of unsaturated fat particularly the presence of double bonds in PUFA was essential for preventing cytokine-mediated endothelial activation, by inhibiting the activation of proinflammatory transcription factors such as nuclear factor κB [45,46]. Although the precise mechanisms through which the PSO-rich meal enhanced endothelial function remain unclear; we might speculate that the potent anti-inflammatory and anti-atherosclerotic effects reported for pucinic acid may be mediating some of the benefits observed in response to this meal. However, we should acknowledge that in addition to the unique fatty acid profile, PSO contains a minor proportion of bioactives, including phytosterols, tocopherols [29], and phenols which have also been shown to promote vascular homeostasis by increasing NO bioavailability and exerting anti-inflammatory effects [47]. Since data relating to the effects of pucinic acid on vascular function are mainly derived from animal and in vitro studies, we cannot discount that differences in the quantities of the individual meal fatty acids, PUFA/SFA ratio or bioactive metabolites between the test oils may also have contributed to the effects observed.

Some studies have associated pomegranate products and punicic acid with beneficial effects on glycemia, particularly in individuals with type 2 diabetes and the metabolic syndrome [48,49]. Consistent with the findings of Rathnayake et al. [15], we found no effects of the meal fat composition on postprandial glycemic measures in postmenopausal females. In contrast, Robertson et al. [14] reported a lower insulin sensitivity after a SFA-rich meal (palm oil and cocoa butter) than meals enriched with unsaturated fatty acids (MUFA, n–6 PUFA, and n–6/n–3 PUFA). Since menopause has been associated with worsening glycemic control and related to altered hormone status and body composition [4], the characteristics of the subject group could have masked the effects of the meal fatty acids on postprandial glucose and insulin responses.

The strengths of this study include the use of test meals that were identical in taste, appearance, and macronutrient composition and the crossover design that carefully accounts for confounders, including exercise. However, this study had certain limitations. The trial involved only postmenopausal female participants, which limits the generalizability of the findings to premenopausal females and middle-aged males. Future studies should explore the acute effects of PSO in other population subgroups and include equal numbers of males and females. A single-blind design was also used which may have introduced bias during data collection and interpretation, as one of the researchers (MMA) was aware of the test oil provided on each study visit. Future studies could include additional blinding of the sample labels during analysis or during the statistical analysis to minimize this potential bias of this study design. Furthermore, formal sample size calculations were not performed for the secondary outcome measures so the significant findings observed require validation in future studies. Likewise, findings from acute postprandial studies conducted in a controlled clinical environment may not be transferable to free-living adults with unrestricted food intakes, with the need to perform longer-term studies to determine whether the transient benefits observed on postprandial TAG, BP and markers of endothelial function after the PSO-rich meal repeated on a daily basis could have implications for overall CVD risk.

In conclusion, this study demonstrates that consuming a single mixed macronutrient meal rich in PSO lowered postprandial TAG concentrations, improved endothelial-dependent microvascular reactivity, and had favorable effects on SBP and sICAM-1 responses. Given that individuals spend a substantial portion of the day in the postprandial state, and non-fasting TAG is an important CVD risk factor in females, consuming PSO may offer a dietary strategy to mitigate menopause-related effects on cardiometabolic health.

## Author contributions

The authors’ responsibilities were as follows – MMA: conducted the research, analyzed the data, conducted the statistical analysis, and wrote the manuscript under the guidance of KGJ, CW, and JPES; CW, JPES: were the supervisors; MMA, KGJ, JPES: designed the human study; KGJ: provided guidance for the sample analysis and during data interpretation with the statistical analysis; and all authors: critically appraised the writing of the manuscript, and read and approved the final manuscript.

## Data availability

The data that support the findings of this study are available from the corresponding author on reasonable request.

## Funding

MMA was supported by a PhD studentship funded by King Abdulaziz University (Saudi Arabia). This research received no specific grant from any funding agency or commercial grant.

## Conflict of interest

The authors report no conflicts of interest.
