# Supplementary material for: Acute Effects of a High-Fat Meal Enriched with Pomegranate Seed Oil on Postprandial Lipemia and Endothelial Function in Postmenopausal Women: a Randomized Controlled Crossover Trial
Source: J Nutr. 2026 Jan 22;156(3):101374. doi: 10.1016/j.tjnut.2026.101374 (PMC13014500; doi:10.1016/j.tjnut.2026.101374)
Supplement: Multimedia component 1 [file mmc1.docx]

**Supplemental Table 1.**  Fatty acid composition of the pomegranate seed oil and control test fat mixtures (g/50 g of fat)

| Fatty Acid | Pomegranate seed oil test meal^1^ | Control oil test meal^2^ |
| --- | --- | --- |
| C_12:0_ | 0.03 | 0.04 |
| C_14:0_ | 0.32 | 0.41 |
| C_16:0_ | 15.36 | 18.89 |
| C_18:0_ | 1.81 | 2.06 |
| C_20:0_ | 0.21 | 0.23 |
| C_22:0_ | 0.06 | 0.03 |
| C_24:0_ | 0.02 | 0.02 |
| C_16:1_ | 0.01 | 0.02 |
| C_18:1 (_*_cis)_* | 15.39 | 18.54 |
| C_20:1 (cis)_ | 0.31 | 0.17 |
| C22:1 | 0.02 | 0.02 |
| C_18:2_ (n-6) | 7.46 | 8.72 |
| C_18:3_ (n-3) | 0.48 | 0.60 |
| C_18:3 (cis9-trans11,cis13)_ Punicic acid | 8.31 | - |

^1^Pomegranate seed oil meal contained 40 g of a mixture of palm oil, rapeseed oil, and safflower oil in a 4:0.5:0.5 ratio, and 10 g of pomegranate seed oil.

^2^Control oil meal contained 50 g of a mixture of palm oil, rapeseed oil, and safflower oil in a 4:0.5:0.5 ratio.

Pomegranate seed oil was analyzed in triplicate for FA composition by GC-MS, as described elsewhere (O’Fallon et al., 2007).

Fatty acid data are based on *Fatty Acids: Supplement to McCance & Widdowson's The Composition of Foods* (R. McCance, E. Widdowson, Royal Society of Chemistry, 1998).

**Reference**

O’Fallon JV, Busboom JR, Nelson ML, Gaskins CT. A direct method for fatty acid methyl ester synthesis: Application to wet meat tissues, oils, and feedstuffs*.* *J Anim Sci*. 2007; 85:1511–21.

**Supplemental Table 2.** Baseline characteristics of participants at the beginning of each acute study visit^1^

| Characteristics | PSO-rich meal | Control meal | P^2^ |
| --- | --- | --- | --- |
| Body weight, kg | 67.3 ± 3.3 | 67.9 ± 3.3 | 0.34 |
| BMI^3^, kg/m^2^ | 24.5 ± 0.9 | 24.8 ± 0.9 | 0.71 |
| Waist circumference, cm | 88.8 ± 2.2 | 88.7 ± 2.4 | 0.88 |
| Hip circumference, cm | 103.4 ± 2.1 | 103.2 ± 2.0 | 0.81 |
| Body fat, % | 35.9 ± 1.9 | 34.5 ± 1.5 | 0.20 |
| Fasting serum biochemical profile | |  |  |
| TC, mmol/L | 5.31 ± .20 | 5.41 ± 0.16 | 0.46 |
| LDL-C^3^, mmol/L | 3.29 ± 0.18 | 3.36 ± 0.13 | 0.71 |
| HDL-C, mmol/L | 1.49 ± 0.07 | 1.52 ± 0.07 | 0.50 |
| TC:HDL ratio | 3.68 ± 0.22 | 3.69 ± 0.22 | 0.89 |
| C-reactive protein^3^, mg/L | 1.51 ± 0.33 | 1.57 ± 0.39 | 0.64 |
| Habitual macronutrient intake |  |  |  |
| Energy, MJ/d | 7.2 ± 0.4 | 7.1 ± 0.4 | 0.36 |
| Total fat, %TE | 36.3 ± 1.5 | 35.9 ± 1.3 | 0.67 |
| SFAs, %TE | 14.3 ± 0.6 | 13.9 ± 0.7 | 0.33 |
| MUFAs, %TE | 12.9 ± 0.9 | 12.6 ± 1.0 | 0.33 |
| n-6 PUFAs^3^, %TE | 5.2 ± 0.4 | 5.7 ± 0.4 | 0.32 |
| n-3 PUFAs^3^, %TE | 0.4 ± 0.1 | 0.9 ± 0.1 | 0.18 |
| TFAs^3^, %TE | 0.5 ± 0.0 | 0.5 ± 0.0 | 1.00 |
| Dietary cholesterol^3^, mg/d | 153 ± 15 | 154 ± 15 | 1.00 |
| Protein, %TE | 13.3 ± 0.6 | 13.9 ± 0.7 | 0.24 |
| Carbohydrates, %TE | 45.0 ± 1.2 | 46.1 ± 1.3 | 0.60 |
| Dietary fiber (AOAC), g/d | 25.3 ± 2.4 | 25.7 ± 2.9 | 0.50 |
| Alcohol^3^, %TE | 2.0 ± 0.7 | 2.1 ± 0.7 | 0.89 |

^1^Values are expressed as unadjusted means ± SEMs; n = 16 for all outcomes. The dietary data was collected using a 3-day unweighed food diary and mean nutrient intakes determined using Nutritics. Abbreviations: Apo, apolipoprotein; HDL-C, high density lipoprotein-cholesterol; LDL-C, low density lipoprotein-cholesterol; PSO, pomegranate seed oil; TC, total cholesterol; TFA, *trans* fatty acid; TE, total energy.

^2^Data were analysed using unpaired t-tests and P<0.05 was considered statistically significant.

^3^Indicates data were log-transformed before analysis

**Supplemental Figure 1**


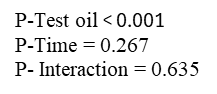

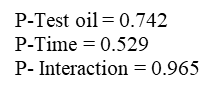


**Supplemental Figure 1:** Postprandial A) acetylcholine-induced (endothelium-dependent) and B) sodium nitroprusside-induced (endothelium-independent) vasodilatory responses in postmenopausal women after consumption of the pomegranate seed oil-rich (open circles) and control (closed squares) meals. Values are expressed as means ± SEMs, n = 16. Linear mixed-model analysis was used to explore the effects of treatment and time, with an adjustment made in all cases for fixed effects of period, time, treatment, age, and BMI. Participant was included as a random effect. *P*≤0.05 was considered statistically significant. Abbreviations: ACh, acetylcholine; LDI, laser Doppler imaging; PU, perfusion units; SNP, sodium nitroprusside.
